# Application of improved graph convolutional network for cortical surface parcellation

**DOI:** 10.1038/s41598-025-00116-0

**Published:** 2025-05-12

**Authors:** Jia Tan, Xiaomei Ren, Yong Chen, Xianju Yuan, Feiba Chang, Rui Yang, Chengqun Ma, Xiaoyu Chen, Miao Tian, Wei Chen, Zihong Wang

**Affiliations:** 1 Department of Medical Engineering, First Affiliated Hospital of Army Medical University, Chongqing, 40039 China; 2 Department of Radiology , First Affiliated Hospital of Army Medical University , Chongqing, 400039 China

**Keywords:** Attention mechanism, Cortical surface parcellation, Deep learning, Graph Convolution network, MRI, Biochemistry, Neuroscience, Biogeochemistry, Health care, Engineering

## Abstract

Accurate cortical surface parcellation is essential for elucidating brain organizational principles, functional mechanisms, and the neural substrates underlying higher cognitive and emotional processes. However, the cortical surface is a highly folded complex geometry, and large regional variations make the analysis of surface data challenging. Current methods rely on geometric simplification, such as spherical expansion, which takes hours for spherical mapping and registration, a popular but costly process that does not take full advantage of inherent structural information. In this study, we propose an Attention-guided Deep Graph Convolutional network (ADGCN) for end-to-end parcellation on primitive cortical surface manifolds. ADGCN consists of a deep graph convolutional layer with a symmetrical U-shaped structure, which enables it to effectively transmit detailed information of the original brain map and learn the complex graph structure, help the network enhance feature extraction capability. What’s more, we introduce the Squeeze and Excitation (SE) module, which enables the network to better capture key features, suppress unimportant features, and significantly improve parcellation performance with a small amount of computation. We evaluated the model on a public dataset of 100 artificially labeled brain surfaces. Compared with other methods, the proposed network achieves Dice coefficient of 88.53% and an accuracy of 90.27%. The network can segment the cortex directly in the original domain, and has the advantages of high efficiency, simple operation and strong interpretability. This approach facilitates the investigation of cortical changes during development, aging, and disease progression, with the potential to enhance the accuracy of neurological disease diagnosis and the objectivity of treatment efficacy evaluation.

## Introduction

**Fig. 1:**
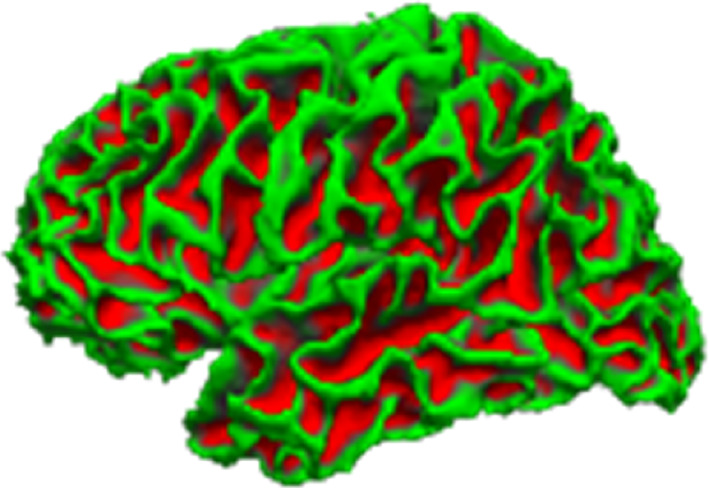
The folds of the cerebral cortex.

The surface of the cerebral cortex plays a vital role in cognition, vision and perception, and is the basis of complex cognitive abilities^1^. Changes in the surface data of the cerebral cortex can reveal new biomarkers and possible relationships with disease processes, which can help in the key steps of early detection, diagnosis, detection, treatment and follow-up of diseases^2–6^. For instance, morphological features including sulcus depth, cortical thickness and surface area can serve as valuable metrics for investigating brain alterations in neuropsychiatric disorders such as Alzheimer’s disease and autism spectrum disorder^2,7–9^. Therefore, researchers are keen to find surface statistical frameworks to study various aspects of the brain^10^. However, its complex folded structure significantly hinders current computational methods for analyzing brain imaging data. As shown in Fig. 1, the complexity of cortical folds and the variability between different subjects make consistent labeling of cortical regions still challenging. Accurate cerebral cortical surface parcellation provides maps of different brain regions, helps to elucidate the functional and structural organization of the brain, and enables effective comparison of results from different studies, which can better understand how the brain works^10^. Not only that, it also reduces data complexity and improves the statistical sensitivity and power of neuroimaging studies^11^. In short, accurate cerebral cortical surface parcellation is the basis for decoding the structural and functional organization of the human brain^12,13^.

The existing methods are mainly divided into two categories: volumetric method and surface method^14^. The volumetric based cerebral cortex parcellation mainly focuses on the volume characteristics of brain tissue, which is very suitable for studying the structure of brain fibers, and is realized by three-dimensional segmentation of brain image data. However, the adjacent voxels in the volume may be far apart on the surface, which ignores the geometric shape of the brain surface to some extent, posing challenges for surface data analysis^15^. Most cortical surface parcellation methods based on the surface method use the idea of atlas registration. First, the surface of the cerebral cortex is iteratively expanded and mapped to the spherical space, then the mapped sphere is segmented using atlas registration or learning labels, and finally the segmentation labels are mapped back to the original space to obtain the segmentation results of the cerebral cortex surface^9,16–20^. This surface-based map registration method^9,21^tends to oversimplify the brain geometry to a topologically equivalent sphere, but there is significant metric distortion between the brain surface and the sphere, which seriously increases the computational burden and reduces the computational efficiency. For example, FreeSurfer^22^, a popular framework for brain analysis, takes 3 ~ 4 h to segment the cortical surface of the cerebral hemisphere, 1 ~ 2 h for spherical mapping, and 1 h for spherical registration. FastSurfer^23^, a fast and accurate deep learning-based neuromedical image processing pipeline. volumetric analysis (1 min on GPU) and cortical thickness analysis (about 1 h) on surface can be performed, which is a good alternative to Freesufer’s processing pipeline. However, its performance depends heavily on the quality and availability of the input data, and the complexity and time consuming of the pre-processing steps can increase the complexity and time cost of the overall workflow.

In addition, traditional methods such as manual labeling^24^or registration-based mapping^25^, which is difficult to learn surface data on intricate surfaces and different objects to find relationships between anatomy and function due to the disadvantages of requiring expert guidance, large computation and low efficiency^26^. The multi-atlas cortical parcellation method^9^accelerated the labeling process through algorithmic consistency. While increasing the number of atlases generally improves performance, this approach has notable limitations. First of all, the computational demands of this method scale with the number of atlases, and the target subject must be aligned with each atlas, making inter-subject registration both inevitable and computationally intensive. Second, these methods often depend on high-quality brain surface reconstructions. Consequently, when high precision is not required, simpler methods based on probabilistic atlases with distortions have been widely adopted^27^. Currently, most studies also utilized cortical surface features to establish surface correspondence^28,29^. In order to consider the topology of gyrus and sulci, Lopez et al. proposed a segmentation method based on the geodesic distance on the surface to create cortical grids and perform k-means clustering^30^. Glasser et al. used a semi-supervised approach to delineate cortical boundaries based on sharp changes in multi-modal MRI data^30^. Machine learning methods are also applied to cortical surface parcellation tasks. Meng et al. first used random forest and automatic context for rough segmentation, and then used graph cutting method to further refine segmentation to improve accuracy and spatial consistency^31^. Although the cortical surface parcellation methods based on atlas registration have been developed very mature, the segmentation performance of these methods is often limited due to the diversity and complexity of the cerebral cortex. There are two main problems in the method of cortical surface parcellation based on map registration. First, establishing correspondence between the atlas and individual subjects is time-consuming, and even optimal spherical registration may not yield optimal segmentation results, as the boundaries of brain regions do not precisely align with geometric features. Second, mapping cortical shapes to segmentation labels requires the design of specialized handcrafted features.

Deep Learning (DL) technology has driven rapid advancements in medical image segmentation due to its powerful learning capabilities. The convolutional neural network (CNN)-based cortical surface parcellation method not only offers significant speed advantages but also enhances the accuracy of cortical surface parcellation. However, traditional CNN techniques cannot be directly applied to cortical surfaces, as a series of complex transformations are required to process this highly folded structure situated in a non-Euclidean domain^32^. In order to solve the above problems, Zhao et al. proposed a novel convolution filter similar to standard convolution on image grids by resampling the cortical surface mapped on the sphere to a uniform geometric structure^20^. This method is mainly inspired by U-Net^33^, extending the classical U-shaped structure from the image domain to the sphere domain, designing the architecture of spherical U-Net, replacing all operations with corresponding spherical operations, and achieving accurate segmentation of the cortical surface. Spherical U-nets are able to learn spatially consistent information in an end-to-end manner without any post-processing. You et al.^34^raised an attention-gated spherical U-Net, a novel deep learning model designed for automated parcellation of the fetal cerebral cortical surface, with potential applications in the early detection of fetal neurodevelopmental disorders. SegRecon^35^, a novel deep learning model for joint reconstruction and segmentation of cortical surfaces, worked directly on MRI volumes and predicts a dense set of surface points and their corresponding segmentation labels. However, the reference map selected for label propagation in this method is sensitive and requires sufficient alignment between the reference map and the same space. SPHARM-Net^36^used a convolutional neural network based on spherical harmonics for vertex cortex segmentation, but this method is still susceptible to absolute spatial differences within a small ROI. Although SPHARM-Net provides fast training and reasoning, spherical mapping is very time consuming. Gopinath et al. put forward a method of learning from MRI volume combined with brain surface reconstruction and segmentation^37^, which is efficient and has accurate topology. The training architecture of this approach is built on a three-dimensional U-Net, volume-based neural network to predict the distance of each voxel to the White Matter (WM), Gray Matter (GM) interface and its corresponding spherical representation in the registry space. The continuous representation of spherical coordinates enables this method to naturally extract implicit surfaces for reconstruction and obtain brain region labels from spherical atlases. Cortical surface registration and segmentation both rely on meaningful representation of cortical features, so they can be jointly optimized by learning shared useful cortical features. To this end, Zhao et al. proposed a deep learning framework for joint cortical surface registration and segmentation using the spherical topology of the cerebral cortex^38^. The network generated segmentation maps based on cortical features of a given cortical surface, while generating individual to atlas deformation fields, and is able to train high-quality segmentation and registration models using less labeled data. This method is more efficient in both registration and segmentation than a network learned alone. When studying longitudinal morphological and functional changes in the human brain, the time consistency and accuracy of the longitudinal cortical surface registration and segmentation are very important. However, most existing methods have been developed for the registration or segmentation of a single cortical surface. Zhao et al.^39^ proposed a semi-supervised learning framework to leverage these inherent relationships from limited labeled data and large amounts of unlabeled data to achieve more robust and time-consistent longitudinal cortical surface registration and parcellation. The above method can achieve fast and accurate segmentation of the spherical surface, but it still takes a long time to map the cortical surface to the spherical surface, and does not utilize the inherent structural information of the original cortical surface. At the same time, deformation from the swelling of the brain surface to a sphere can cause distortion, and if there are topological defects on the original surface, such as holes or triangles flipped, it is sometimes difficult to achieve accurate spherical mapping. What’s more, most of the existing cortical surface parcellation models need to adopt the graph cutting method or Markov random field for post-processing to optimize the segmentation results.

Regular grid structure, which has consistent neighborhood relations in Euclidean space, provides the basis for CNN model and makes CNN achieve great success in feature learning on 2D/3D images in Euclidean space^40^. However, the shapes of many structures in medical images have an inherent spherical topology in manifold space, such as the surface of the cerebral cortex represented by a triangular grid. Due to the lack of a consistent neighborhood definition for cortical surface data, segmenting it rapidly and accurately using CNN remains a significant challenge. Graph Neural Network (GNN) is a powerful tool for learning geometric features of non-Euclidean structured data and has emerged as a prominent field in machine learning within just a few years^41^. Cucurull et al. applied Graph Convolutional Networks (GCNs) and Graph Attention Networks (GATs) to cortical surface parcellation^42^, addressing the challenge of dividing the cerebral cortex into functionally discrete regions. This method leverages the underlying structure of the data, demonstrating superior performance compared to node feature-based multilayer perceptron approaches. However, it has only been applied to parcellate a limited number of regions, and further research is required to extend its application to the entire cortical surface. He et al. proposed the spectral graph transformer Networks^43^, a new method for learning the transformation matrices required to align brain networks using a direct data-driven approach. The transformation used very few random sub-sampling nodes in the spectral domain to learn the alignment matrix of multiple brain surfaces and map the input spectral coordinates to the reference set. ASEGAT^44^performed cortical parcellation directly in the original grid space. While this strategy enables accurate and efficient parcellation of cortical surfaces, it is highly sensitive to the quality of the surface reconstruction process. For instance, minor errors or holes in the reconstructed cortical mesh can lead to failures in the segmentation task when using graph convolution methods. In the task of cortical surface parcellation, we opt for GNNs over CNNs based on the following considerations: (1) The cortical surface is a complex non-Euclidean structure (e.g., mesh or manifold) with inconsistent vertex counts and connectivity, whereas CNNs are primarily designed to handle Euclidean data (e.g., fixed-size images). (2) When processing cortical surfaces, CNNs typically require projecting the surface data onto a 2D plane (e.g., spherical projection), which may lead to the loss or distortion of topological information. In contrast, GNNs can operate directly on the cortical surface mesh, preserving the topological structure and avoiding information loss caused by projection. (3) The morphological variability of cortical surfaces across individuals is significant, and CNNs may have difficulty directly handling such variability. GNNs, however, can flexibly adapt to the cortical surfaces of different individuals through graph structures without requiring strict alignment or normalization. Despite the notable advantages of GNNs in cortical surface segmentation, their application still faces several challenges. For instance, existing methods often fail to fully leverage global information, such as the proximity relationships between brain regions, which may lead to anatomically abnormal segmentation^45^. Additionally, the interpretability of GNNs in clinical settings remains insufficiently addressed^46^, limiting their broader adoption in medical applications. Therefore, future research should focus on enhancing the ability of GNNs to integrate global information and improving their interpretability to better meet the demands of cortical surface segmentation tasks.

**Fig. 2:**
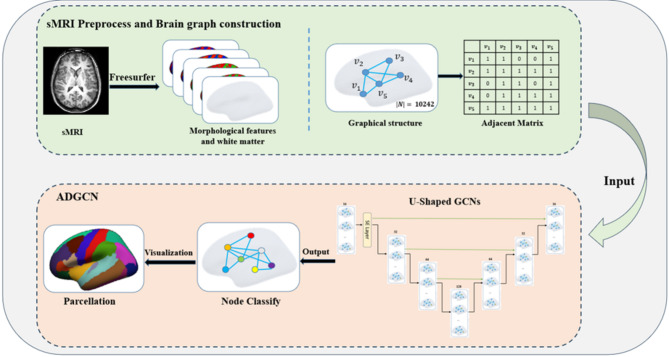
GNN-based flow chart of cerebral cortex surface parcellation.

It is very important to accurately segment the surface of the cerebral cortex, which has a complex folded structure, into anatomically and functionally significant regions. Therefore, an attention-mechanic-based Deep Graph Convolutional network for cerebral cortex surface parcellation was proposed, and its process was shown in Fig. 2. Firstly, FreeSurfer software was used to preprocess sMRI images, obtain WM surface and morphological features build a brain graph according to the WM surface vertex connection relationship, splicing the brain graph and features, and then input it into the network, assign labels to each node in the brain graph, and finally cortical surface parcellation result is visualize by FreeSurfer. The main contributions of this study are as follows:

- A deep graph convolutional network based on attention mechanism is proposed, which directly performs on the cortical surface, and can achieve accurate and fast end-to-end parcellation.
- The deep convolutional layer of the symmetrical U-shaped structure can effectively convey the detailed information related to the original brain graph, which helps the network enhance the feature extraction ability and effectively learn the complex structure of the brain graph;
- Introduced the Squeeze and Excitation module, which makes the network better able to capture key features, suppress unimportant features, and improve network parcellation performance;

The U-shaped GCN architecture was adopted primarily due to the widespread success of U-Net^33^ in medical image segmentation tasks and its exceptional capability for multi-scale feature extraction. The encoder-decoder structure of U-Net, augmented by skip connections, effectively integrates features across different hierarchical levels, enabling the simultaneous capture of local details and global contextual information. This capability is particularly critical for the accurate processing of cortical surface data, which often exhibits complex geometric structures and fine-grained features. Therefore, we utilized a U-shaped GCN architecture as the foundational framework to address the requirements of cortical surface parcellation tasks.

## Methods

A. *Graph Convolutional Network*.

**Fig. 3:**
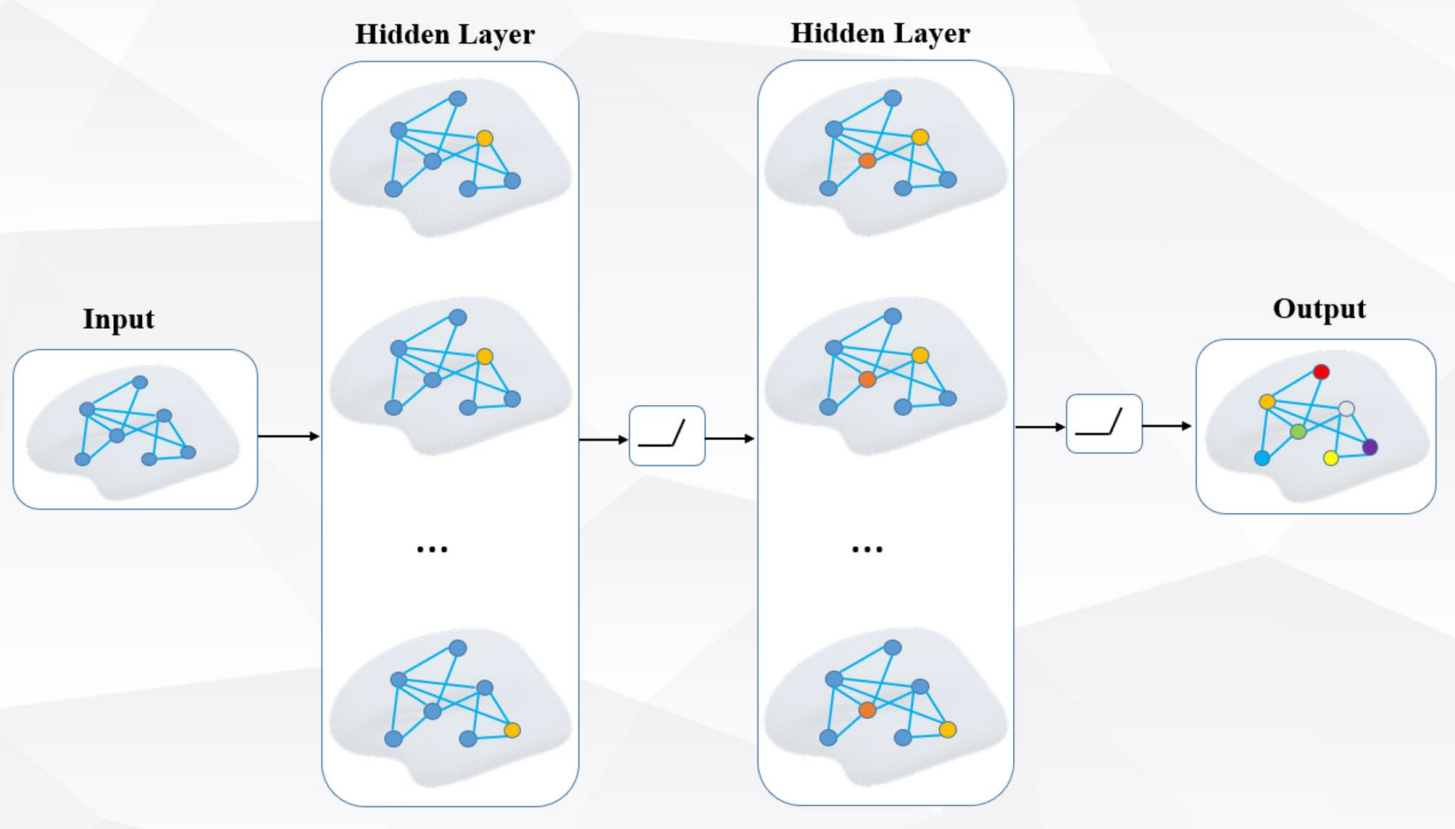
Two-layer graph convolution layer structure (Reprinted with permission from Ref.^55^).

GCN is designed to explicitly take advantage of the underlying graph structure of the brain and operate directly on the graph. GCN calculates the product between the input data and the filter in the frequency domain (i.e. the Fourier domain) to derive the spectral convolution inherent in the graph structure. GCN consists of multilayer graph convolution, that is, first order local approximation of spectral convolution. Each graph convolution layer is regarded as the processing of the information of the first-order adjacent nodes, and the information aggregation and transformation of multi-order nodes are realized by superimposing multiple layers, and a more advanced and abstract feature representation is gradually extracted, so as to better capture the complexity and structural information of the graph data. This study uses GCN as the baseline model, and its propagation rules are as follows:

1 $$\:\begin{array}{c}{H}^{\left(l+1\right)}=\sigma\:\left({\stackrel{\sim}{D}}^{-\frac{1}{2}}\stackrel{\sim}{A}{\stackrel{\sim}{D}}^{-\frac{1}{2}}{H}^{\left(l\right)}{W}^{\left(l\right)}\right)\end{array}$$

where, $\:A$ is the adjacency matrix of each subject’s cortical surface and $\:\stackrel{\sim}{A}=A+{I}_{N}$ is the undirected graph $\:\mathcal{G}$ adjacency matrix with the relationship of each node on the cortical surface added. $\:{I}_{N}$ is the identity matrix, which is expressed as each node has an edge connected with itself, and the identity matrix is added in order to retain the characteristic information of the vertex itself when information is transmitted. If not add the identity matrix, then only the features of the neighbor nodes are aggregated. $\:{\stackrel{\sim}{D}}_{ii}=\sum\:_{j}{\stackrel{\sim}{A}}_{ij}$, $\:{W}^{\left(l\right)}$ is a trainable weight matrix of a specific layer, $\:\sigma\:(\cdot\:)$ represents the activation function, $\:{H}^{\left(l\right)}\in\:{\mathbb{R}}^{N\times\:D}$ is the feature matrix of the $\:l$-th layer, the features of each node as the most original input features, that is $\:{\text{H}}^{\left(0\right)}=\text{X}$. The form of this propagation rule can be obtained by first order approximation of the local spectral filter on the graph. The simple two-layer GCN model is shown in Fig. 3:

Spectral convolution on graph defined as a signal $\:x\in\:{\mathbb{R}}^{N}$ (scalar per node) having a filter $\:{g}_{\theta\:}=diag\left(\theta\:\right)$ in the Fourier domain of $\:\theta\:\in\:{\mathbb{R}}^{N}$:

2 $$\:\begin{array}{c}{g}_{\theta\:}*x=U{g}_{\theta\:}{U}^{T}x\end{array}$$

where, $\:x$ is the eigenvector of the node in graph, $\:U$ is the eigenvector matrix of the normalized graph Laplacian matrix $\:L={I}_{N}-{D}^{-\frac{1}{2}}A{D}^{-\frac{1}{2}}=U{\Lambda\:}{U}^{T}$, and its diagonal matrix $\:{\Lambda\:}$ and $\:{U}^{T}x$ are the Fourier transform of the graph. $\:{g}_{\theta\:}$ that can be understood as eigenvalues of $\:L$, for example $\:{g}_{\theta\:}\left({\Lambda\:}\right)$. The cost of calculating (2) is high because the complexity of multiplying it with the eigenvector matrix $\:U$ is $\:\text{{\rm\:O}}\left({N}^{2}\right)$. In addition, the computed feature decomposition of $\:L$ can be very expensive for large graphs, so it needs to be further improved. In order to generate spatial local filters and eliminate the need to compute eigenfactorization of the Laplacian matrix of a normalized graph, it has been proposed that $\:{g}_{\theta\:}\left({\Lambda\:}\right)$ can be approximated well to order $\:k$ by truncation expansion of the Chebyshev polynomial $\:{T}_{k}\left(x\right)$^47^. The principle of Chebyshev polynomial is as follows:

3 $$\:\begin{array}{c}{T}_{0}\left(x\right)=1;{T}_{1}\left(x\right)=x;{T}_{k}\left(x\right)=2x{T}_{k-1}\left(x\right)-{T}_{k-1}\left(x\right)\end{array}$$

So as to achieve computation localization and reduce computational complexity, the improved convolution kernel is as follows:

4 $$\:\begin{array}{c}{g}_{{\theta\:}^{{\prime\:}}}\left({\Lambda\:}\right)\approx\:\sum\:_{k=0}^{K}{\theta\:}_{k}^{{\prime\:}}{T}_{k}\left(\stackrel{\sim}{{\Lambda\:}}\right)\end{array}$$

where, $\:\stackrel{\sim}{{\Lambda\:}}=\frac{2}{{\lambda\:}_{max}}{\Lambda\:}-{I}_{N}$, $\:{\lambda\:}_{max}$ is the maximum eigenvalue of $\:L$,$\:\:{\theta\:}^{{\prime\:}}$ is a vector of the Chebyshev coefficient. Thus, the convolution of signal $\:x$ and filter $\:{g}_{{\theta\:}^{{\prime\:}}}$ as follows:

5 $$\:\begin{array}{c}{g}_{{\theta\:}^{{\prime\:}}}*x\approx\:\sum\:_{k=0}^{K}{\theta\:}_{k}^{{\prime\:}}{T}_{k}\left(\stackrel{\sim}{L}\right)x\end{array}$$

where $\:\stackrel{\sim}{L}=\frac{2}{{\lambda\:}_{max}}\text{L}-{I}_{N}$, $\:{\left(U{\Lambda\:}{U}^{T}\right)}^{k}=U{{\Lambda\:}}^{k}{U}^{T}$. Note that (5) is a polynomial of order $\:K$ in the Laplace operator, i.e., it depends only on the neighbor node that is the largest $\:K$ steps away from the central node. Regardless of the convolution formula, it ultimately simplifies the parameters to $\:K$ and eliminates the need for feature decomposition, directly transforming with the Laplacian matrix $\:L$. The computational complexity is greatly reduced.

In this linear formula for GCN, which can be further approximated $\:{\lambda\:}_{max}\approx\:2$, the neural network parameters will adapt to changes of this scale during training.

6 $$\:\begin{array}{c}{g}_{{\theta\:}^{{\prime\:}}}\star\:x\approx\:{\theta\:}_{0}^{{\prime\:}}x+{\theta\:}_{1}^{{\prime\:}}\left(L-{I}_{N}\right)x={\theta\:}_{0}^{{\prime\:}}x-{\theta\:}_{1}^{{\prime\:}}{D}^{-\frac{1}{2}}A{D}^{-\frac{1}{2}}x\end{array}$$

There are two free parameters $\:{\theta\:}_{0}^{{\prime\:}}$ and $\:{\theta\:}_{1}^{{\prime\:}}$ the filter parameters can be shared across the graph. The continuous application of this form of filter can then efficiently convolve the $\:k$ order neighborhood of the nodes, where $\:k$ is the number of continuous filtering operations or convolution layers in the neural network model. Limiting the number of parameters in the network model can solve the overfitting problem and reduce the computational complexity. Therefore, the above formula can be simplified as:

7 $$\:\begin{array}{c}{g}_{{\theta\:}^{{\prime\:}}}\star\:x\approx\:\theta\:\left({I}_{N}+{D}^{-\frac{1}{2}}A{D}^{-\frac{1}{2}}\right)x\end{array}$$

There is a single parameter $\:\theta\:={\theta\:}_{0}^{{\prime\:}}=-{\theta\:}_{1}^{{\prime\:}}$ Note that the eigenvalues of $\:{I}_{N}+{D}^{-\frac{1}{2}}A{D}^{-\frac{1}{2}}$ in the range [0,2]. Therefore, when the operator is used in a deep neural network model, repeated application of the operator can lead to numerical instability and gradient explosion or disappearance. To alleviate this problem, renormalization techniques is introduced: $\:{I}_{N}+{D}^{-\frac{1}{2}}A{D}^{-\frac{1}{2}}\to\:{\stackrel{\sim}{D}}^{-\frac{1}{2}}\stackrel{\sim}{A}{\stackrel{\sim}{D}}^{-\frac{1}{2}}$, $\:\stackrel{\sim}{A}=A+{I}_{N}$, $\:{\stackrel{\sim}{D}}_{ii}=\sum\:_{j}{\stackrel{\sim}{A}}_{ij}$. A two-layer GCN is considered for semi-supervised node classification on a graph with symmetric adjacency matrices, binary or weighted. We calculates $\:\widehat{A}{=\stackrel{\sim}{D}}^{-\frac{1}{2}}\stackrel{\sim}{A}{\stackrel{\sim}{D}}^{-\frac{1}{2}}$ in the pre-processing step, which makes the previous model more concise:

8 $$\:\begin{array}{c}Z=f\left(X,A\right)=softmax\left(\widehat{A}\text{R}\text{e}\text{L}\text{U}\left(\widehat{A}X{W}^{\left(0\right)}\right){W}^{\left(1\right)}\right)\end{array}$$

where, $\:{W}^{\left(0\right)}\in\:{\mathbb{R}}^{C\times\:H}$ is the weight matrix from the input layer to the hidden layer, which has H feature map. $\:{W}^{\left(1\right)}\in\:{\mathbb{R}}^{H\times\:F}$ is the weight matrix from the hidden layer to the output layer.

GCN-Chebyshev may improve the generalization performance of the model to some extent through the Chebyshev polynomial approximation filter, because it can deal with different graph structures and feature distributions more flexibly. However, flexibility can also introduce additional model complexity and computational overhead, so we chose GCN as the baseline model. Considering that the number of nodes in the input graph is still very large, residual connections and batch normalization are added to the GCN to solve the problem of overfitting, thereby improving its learning ability of the intrinsic geometric context of the original cortical surface.

B. *Graph-based Squeeze-and-Excitation Module*.

**Fig. 4:**
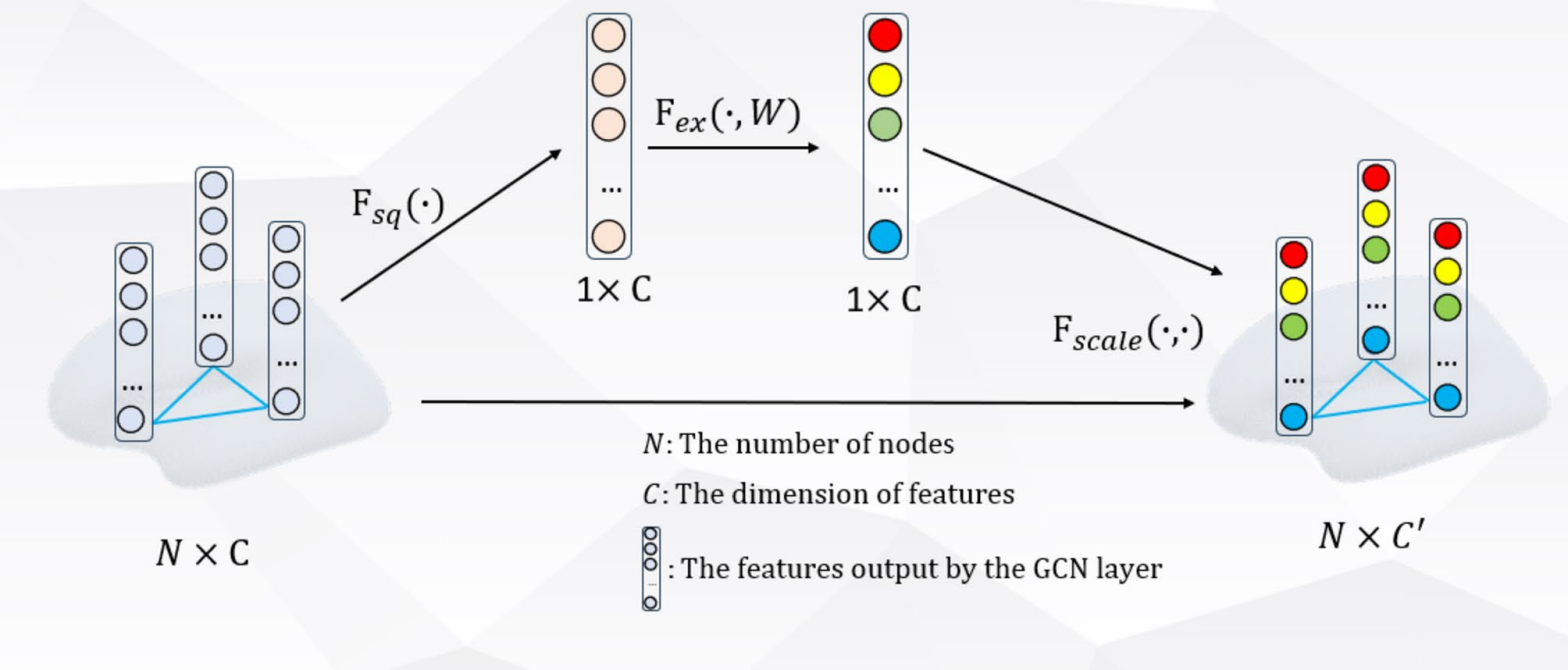
Graph-based Squeeze-and-Excitation Module.

In cognitive science, due to the bottleneck of information processing, models with attention mechanisms selectively focus on specific parts of input information while ignoring other less relevant information, enabling networks to efficiently process complex information environments. Currently, many studies employ squeeze-and-excitation^48^modules to enhance feature extraction^44,49^. To this end, we integrate an SE layer after the first GCN layer, which can explicitly model the interdependencies between convolutional feature channels. SE module gives more weight to important features and inhibits less important features, which can realize feature recalibration and improve expression ability. The SE module consists of a global average pool and two fully connected (FC) layers that capture inter-channel information and dynamically adjust the weight of the feature map for end-to-end training, as shown in Fig. 4. First, the global features are obtained using the squeeze operation $\:{F}_{sq}$, and then the importance between the channel features is learned through the excitation operation $\:{F}_{ex}$ to obtain different feature responses. Finally, the learned excitation value is multiplied with the original feature to obtain the final feature, so as to realize the adaptive adjustment of the feature channel according to the weight coefficient, enhance the features that are conducive to segmentation, and suppress the unimportant features.

Here, we take the output of the graph convolutional network as the input to the SE module. Since convolution is only operated in local space (without global sensitivity field), it is difficult to obtain enough information to extract the relational features between channels. Therefore, the squeeze operation $\:{\mathbf{F}}_{sq}$ is used to obtain the global information embedding of each channel through the operation of global average pooling. Formally, a compression vector $\:\mathbf{z}\in\:{\mathbb{R}}^{C}$ is generated by shrinking the spatial dimension $\:\mathbf{h}\in\:{\mathbb{R}}^{N\times\:C}$ of the input feature graph, and the formula for calculating the c-th element is as follows:

9 $$\:\begin{array}{c}{z}_{c}={\mathbf{F}}_{sq}\left({\mathbf{h}}_{c}\right)=\frac{1}{N}\sum\:_{i=1}^{N}{\mathbf{h}}_{c}\left(i\right),\:z\in\:{\mathbb{R}}^{C}\end{array}$$

where $\:N$ is the number of nodes, $\:C$ is the feature dimension.

The squeeze operation has obtained the global description features, so the next step is to learn the channels, that is, the relationships between the features. Therefore, excitation operations $\:{F}_{ex}$ is used to learn the weights of each channel to excite important features and suppress non-essential features. In order to reduce the complexity of the model, fit the complex correlation between each channel and improve the generalization ability of the model, a splicing structure with two FC layers are adopted.

The first FC layer is called the dimensionality reduction layer, and the number of neurons will be much less than the number of input channels. By setting the scaling parameter r, the layer reduces the number of input channels from the original c to c/r, which is achieved by mapping the higher-dimensional eigenvector to a lower-dimensional space. The dimensionality of the output feature vector is reduced, which helps to remove redundant information, thus reducing the complexity of subsequent calculation and improving the generalization ability of the model. The second FC layer, called the recovery layer, restores the output channel c/r of the first FC layer to the original channel number c, and assigns an appropriate weight value to each channel so that it can be multiplied by channel with the original feature map to achieve the weighting of different channel features. Normalized weights between 0 and 1 are obtained using a gating mechanism in the form of sigmoid:

10 $$\:\begin{array}{c}s={\mathbf{F}}_{ex}\left(\mathbf{z},\mathbf{W}\right)=\sigma\:\left(g\left(\mathbf{z},\mathbf{W}\right)\right)=\sigma\:\left({\mathbf{W}}_{2}\text{R}\text{e}\text{L}\text{U}\left({\mathbf{W}}_{1}\varvec{z}\right)\right)\end{array}$$

where $\:{\mathbf{W}}_{1}\in\:{\mathbb{R}}^{\frac{C}{r}\times\:C}$, $\:{\mathbf{W}}_{2}\in\:{\mathbb{R}}^{C\times\:\frac{C}{r}}$ are the weight of two FC layers to reduce and restore dimensions, $\:r$ is a scaling parameter, $\:\sigma\:$ represents sigmoid function. When using SE Block, make sure that the number of channels and the reduction ratio of the input feature are set reasonably to avoid excessive computational overhead, so we will set it to 4. The graph-based SE module is to multiply the weights s of each channel learned by the squeeze excitation operation and the original features h to adjust the weights, making the model more discriminative of the features of each channel:

11 $$\:\begin{array}{c}{\stackrel{\sim}{\mathbf{h}}}_{c}={\mathbf{F}}_{scale}\left({\mathbf{h}}_{c},\:{s}_{c}\right)={s}_{c}\bullet\:{\mathbf{h}}_{c}\end{array}$$

where $\:{\stackrel{\sim}{\mathbf{h}}}_{c}$ is the output of SE module, $\:{\mathbf{F}}_{sclae}({\mathbf{h}}_{c},{s}_{c})$ refers to the channel multiplication between scalar $\:{s}_{c}$ and feature graphs $\:{\mathbf{h}}_{c}\in\:{\mathbb{R}}^{N}$.

The graph-based SE module can improve the quality of cortical surface parcellation by making the model pay more attention to the important features and suppress the unimportant ones. In addition, the module is lightweight and efficient, making it flexible to port to other network architectures.

## Experiments

C. *Mindboggle Datasets*.

This study uses the Mindbogle-101 dataset^24^, the world’s largest and most complete set of free, publicly accessible, manually labeled images of the human brain. It contains 101 subjects from various place with a cortical grid range of 102 ~ 185 K vertices, the details of which are shown in Table 1. All the data and related software and updates are available at http://mindboggle.info/data.

**Table 1:** Mindboggle-101 datasets.

Subject	Number	Age (mean ± std)	Sex (male + female)
NKI-RS-22	22	20–40(26.0$\:\pm\:$5.2)	12 + 10
NKI-TRT-20	20	19–60(31.4$\:\pm\:$11.1)	14 + 6
MMRR-21	21	22–61(31.8$\:\pm\:$9.2)	11 + 10
MMRR-3 T7 T-2	2	22, 24	2 + 0
HLN-12	12	23–39(27.8$\:\pm\:$4.6)	6 + 6
OASIS-TRT-20	20	19–34(23.4$\:\pm\:$3.9)	8 + 12
Colin27-1	1	33	1 + 0
Twins-2	2	41	2 + 0

It is worth to note that the Mindboggle-101 dataset does not provide manual segmentation labels for the OASIS-TRT-20-1 subject, so we only used complete data for 100 subjects in the experiment. The Mindboggle-101 dataset, which includes 31 cortical regions, was manually labeled for each subject’s left and right hemispheres according to the DKT protocol. For comparison with other methods, we use the left brain label (lh.labels.DKT31.manual.annot) as ground truth, which is the gold standard for cortical surface parcellation.

D. *Data Preprocessing*.

**Fig. 5:**
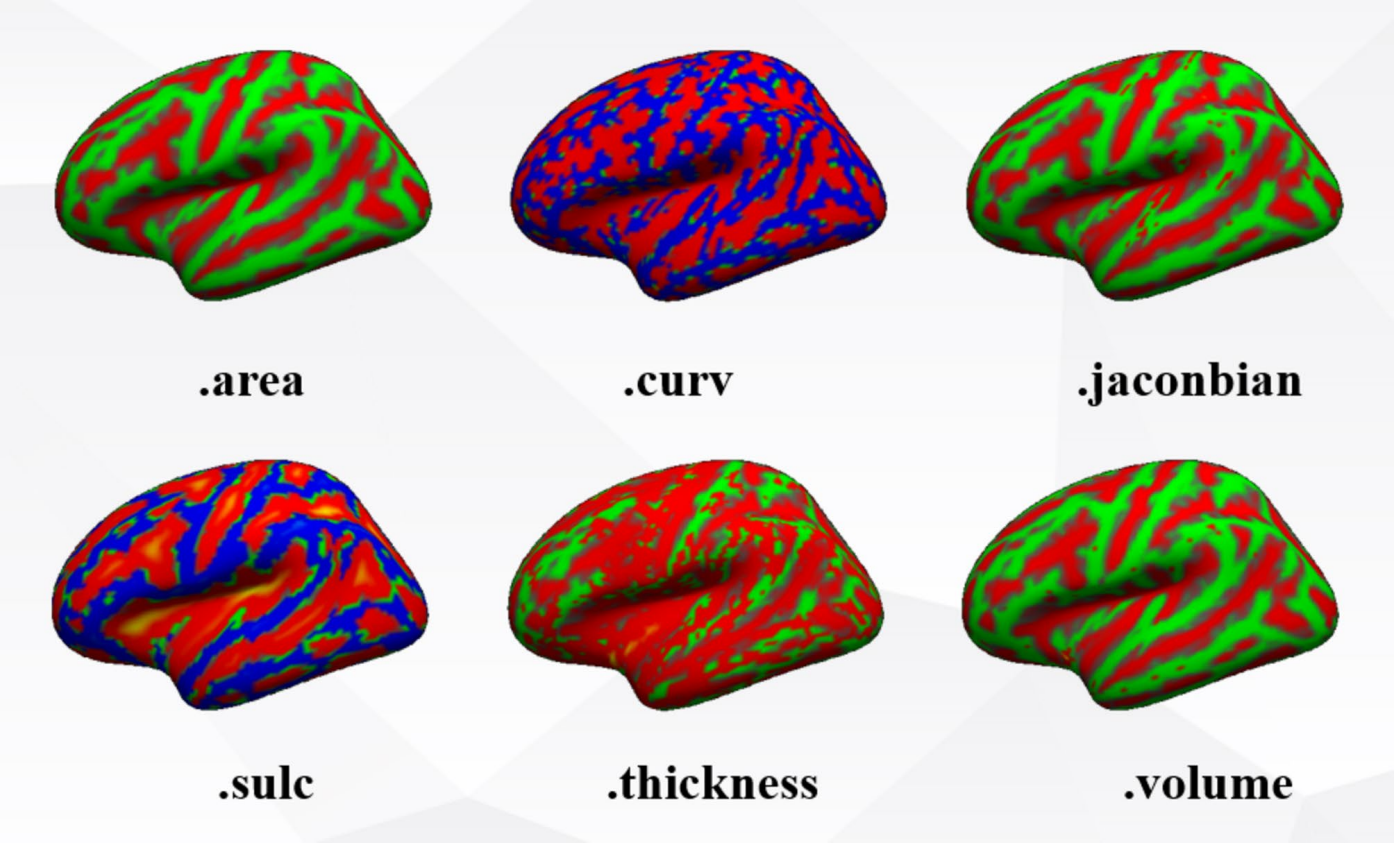
Six morphological features of the surface of the cerebral cortex.

The six morphological features of each subject’s WM surface and each vertex were further used for the construction of the brain map. Cortical segmentation is the assignment of an anatomical label to each vertex of the cortical surface. The statistics of cortical morphological features are shown in Fig. 5. The surface area of the cortex is calculated by taking the current grid point as the center and calculating the average of the plane area formed by all the other grid points which are topologically connected to the grid point. The mean curvature is calculated as 1/r, where r is the radius of the tangent circle at the corresponding grid point, which can be used to reflect the degree of fold of the cortex. The Jacobi measure reflects the displacement and deformation of the sample data relative to the brain template during the reconstruction of the cerebral cortex grid surface. The calculation of sulci depth should first determine the datum level. The criterion for selecting the datum level is that the algebraic sum of the distance between all grid points and the datum level is zero, and the sulci depth is the vertical distance between the outer surface of the sulci and the inner surface of the gyri and the datum level, wherein the sulci depth value of the gyri is positive and the sulci depth value of the sulci is negative. Cortical thickness, to a certain extent, represents the development and aging of brain tissue, and is also associated with neurological decline and other diseases. Cortical thickness is the shortest distance between the inner surface of the brain (the interface between GM and WM) and the outer surface (the interface between GM and CSF). Measurement distortion reflects the differences that occur in the individual brain relative to the average brain template cortical surface, i.e., displacement and deformation, which can lead to misunderstandings about the structure and function of the cortex, thus affecting the accuracy and reliability of the study results. The volume is calculated by first finding all the triangles composed of other grid points near the grid point, then calculating the volume of the grid point and the tetrahedron composed of each triangle and adding it together. The volume of each small tetrahedron is calculated by multiplying the area of the triangle and the distance from the grid point to the triangle.

**Fig. 6:**
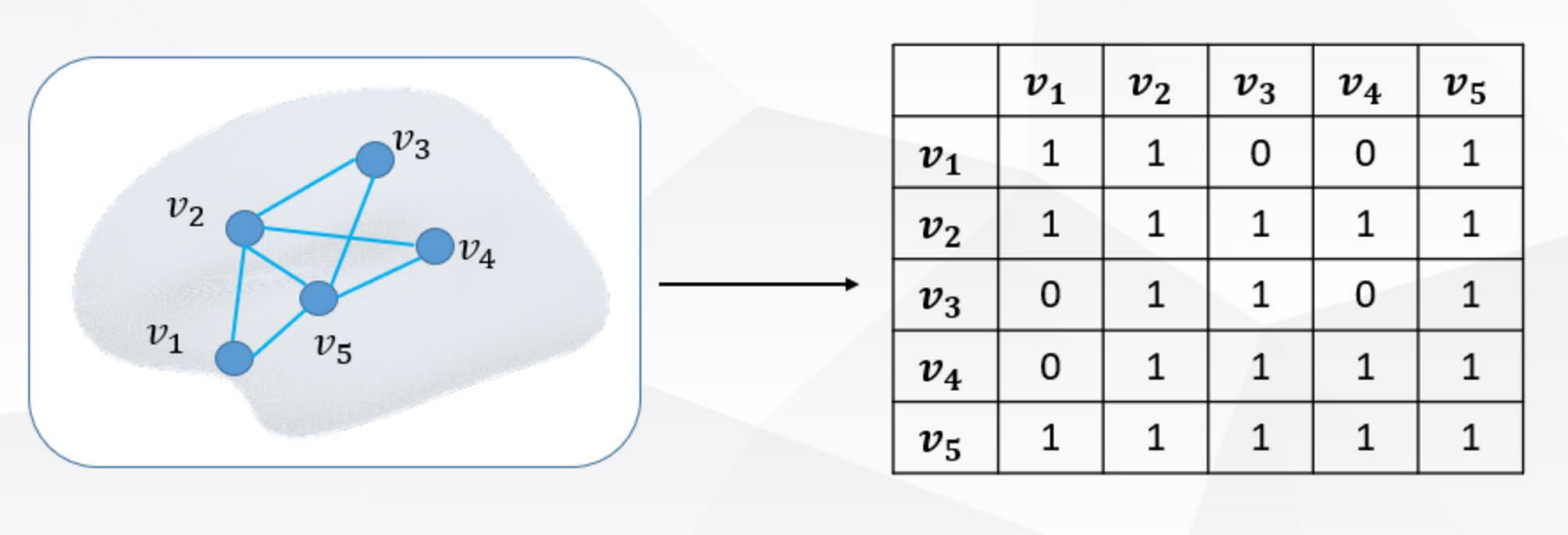
Brain graph construction.

Since GCN is used for surface of the cerebral cortex parcellation, the WM surface represented by the triangular grid needs to be used to construct the graph - where the vertices of the grid are regarded as graph nodes and the edges of the grid are regarded as graph edges. The basis for constructing the brain map is shown in Fig. 6. In this study, each vertex on the surface of the cerebral cortex is taken as the node in the graph structure, and the connection relationship between the vertices is taken as the edge, according to which the adjacency matrix is constructed, that is, the undirected graph of the cerebral cortex surface. Vertex $\:{v}_{1}$ is directly connected to $\:{v}_{2}$, $\:{v}_{3}$, $\:{v}_{4}$, $\:{v}_{5}$, and are represented in the adjacency matrix $\:A$ on the right, where the value of position $\:{A}_{{(v}_{1},{v}_{2})}$, $\:{A}_{({v}_{1},{v}_{3})}$, $\:{A}_{({v}_{1},{v}_{4})}$, $\:{A}_{({v}_{1},{v}_{5})}$ are set to 1. Since the adjacency matrix has the property of complete symmetry, the value of position $\:{A}_{\left({v}_{2}{,v}_{1}\right)}$, $\:{A}_{({v}_{3},{v}_{1})}$, $\:{A}_{({v}_{4},{v}_{1})}$, $\:{A}_{({v}_{5},{v}_{1})}$ are set to 1, and the remaining positions are set to 0. In order to introduce the characteristics of the node itself, it is necessary to add a self-ring to each node, that is, set the value of $\:{A}_{({v}_{1},{v}_{1})}$ to 1, and so on for the remaining nodes.

In order to ensure adequate resolution and uniform number of delimited cortical surfaces, all cortical surfaces were resampled to fsaverage5 cortical spatial coordinates, which are order 5 icosahedrons with 10,242 vertices, which alleviates the problems of data redundancy, inefficiency, and lack of memory caused by large maps. Please note that the input data in the following experiments are all processed morphological features and brain graph of each subject.

E. *Evaluation Metrics*.

Since Dice coefficient can accurately measure the performance of the model in various categories, and accuracy can quickly reflect the overall prediction accuracy of the model, we use Dice coefficient and accuracy as evaluation indexes to quantify the parcellation performance of the cortex surface. Dice coefficient is an index to measure the degree of overlap between the predicted results and the real labels. The segmentation results can be evaluated by calculating the ratio of intersection and union, so as to reflect the performance of the model in various categories more accurately. Accuracy is the ratio of the number of vertices correctly predicted to the total number of target vertices. The value range of the two indicators is [0,1], the parcellation performance is 1 at best and 0 at worst. Dice coefficient and precision are defined as follows:

12 $$\:\begin{array}{c}\text{D}\text{i}\text{c}\text{e}\left(\text{G,P}\right)\text{=}\frac{{2|G \cap P|}}{\left|\text{G}\right|\text{+|P|}}\end{array}$$

13 $$\:\begin{array}{c}\text{A}\text{c}\text{c}\text{u}\text{r}\text{a}\text{c}\text{y}\left(\text{G,P}\right)\text{=}\frac{{|G\cap P|}}{\text{|G|}}\end{array}$$

where G is the true label and P is the predictive label, $\:\cap\:$ representing overlap and $\:|\bullet\:|$ is the number of vertices in the region.

F. *Implement Details*.

This paper still considers basic, simple architectures and training strategies to verify the effectiveness of ADGCN networks for cortical surfaces parcellation. In this study, all subjects were trained and tested with 5-fold cross-validation across the entire dataset. Due to the large number of nodes in each brain graph ($\:N$=10242), based on the limitation of memory size, batch size is set to 1 in this paper. In addition, set the learning rate to 0.01 and dropout to 0.1. The model uses Cross Entropy Loss and L2 Loss as the total loss function for training, and the node input features are standardized. In this case, good verification performance is achieved. For deep networking, we therefore use residual connection and batch normalization in each layer of GCN. In this paper, ADGCN is trained with Adam optimizer^50^, the whole training process has 200 epochs, and Tensorflow1.15.0 is implemented on Ubuntu server and NVIDIARTXTITANGPU. Public access to our code and data are available at https://github.com/tanjia123456/ADGCN/.

## Results and discussion

In this study, the GCN model with BN and residual connections is used as the benchmark model. Table 2 shows the ablation experiment of the effect of SE modules and the number of hidden layers on the parcellation performance. The number of GNN layers has a significant impact on network performance. Generally, shallow GNN can capture the local features of the graph, and the calculation efficiency is high, so the GNN of 2 to 3 layers has been able to achieve better performance in many applications. In the original model, two-layer GCN was used, probably because the two-layer GCN can aggregate the feature information of most nodes in the network to the target node. If the layer of network got deeper, the object of each node gathering features will be a large number of repeats, resulting in the trained embedding ability is weak. However, you can see that GNN captures more global features and has better segmentation performance as the number of layers increases in Table 2. For example, when the number of hidden layers is 7 and the output feature dimensions of each hidden layer are 16,32,64,128,64,32,16, the ADGCN obtains 86.58% dice coefficient and 86.57% accuracy. Compared with the model which only use one hidden layer, dice and accuracy are improved by 10.85% and 6.36%, respectively. We believe that the optimal number of layers of GCN may be related to the sparsity of the neighbor matrix of the constructed graph, and the sparsity of the brain graph is higher, so the deep network can learn features better. Similarly, the ASEGAT network uses only two layers of GAT, but uses multiple attention heads, and its performance is significantly improved by increasing the number of attention heads. In addition, we use a symmetrical U-shaped structure in ADGCN, which enables it to efficiently convey detailed information related to the original brain map, which helps to improve the accuracy of segmentation. At the same time, thanks to batch normalization and residual connection, it avoids a series of problems such as gradient disappearance, over smoothing and overfitting caused by representation vector convergence, which enables the learning task to continue. In GCN, each node contains rich feature information, and different feature channels may have different importance for node classification. By introducing a channel attention mechanism, GCN is able to better capture these key features, thereby improving overall performance. We also discussed the impact of different reduction ratio on the parcellation performance in Table 2, and found that ratio = 4 was more reasonable, resulting in 76.84% dice coefficient and 81.34 accuracy%. Compared with ADGCN (hidden layer = 1), the model with SE module added produced a modest improvement in performance, which dice and accuracy are improved by 1.11% and 1.13%, respectively. In the end, we found that setting the number of hidden layers is set to 7 and introducing the SE module achieved better performance, with dice and accuracy of 88.53% and 90.27%, respectively.

**Table 2:** The effect of the number of SE modules and hidden layers on parcellation performance.

Methods	SE module	Hidden Layer (Number of units)	Dice (%)	Accuracy (%)
ADGCN	No	1 (16)	75.73	80.21
		2 (16, 32)	83.00	84.91
		3 (16, 32, 64)	79.86	79.81
		4 (16, 32,…,128)	82.63	83.87
		5 (16, 32,…,64)	84.29	85.38
		6 (16, 32,…,32)	80.87	81.29
		7(16, 32,…,16)	86.58	86.57
ADGCN	Yes (*r* = 2)	1 (16)	71.13	72.80
	Yes (*r* = 4)		76.84	81.34
	Yes (*r* = 8)		70.87	74.47
	Yes (*r* = 16)		71.76	75.59
	Yes (*r* = 32)		75.68	78.96
	Yes (*r* = 64)		75.14	76.37
ADGCN	Yes (*r* = 4)	7(16, 32,…,16)	88.53	90.27

Note: r represents the reduction ratio in the SE module.

**Fig. 7:**
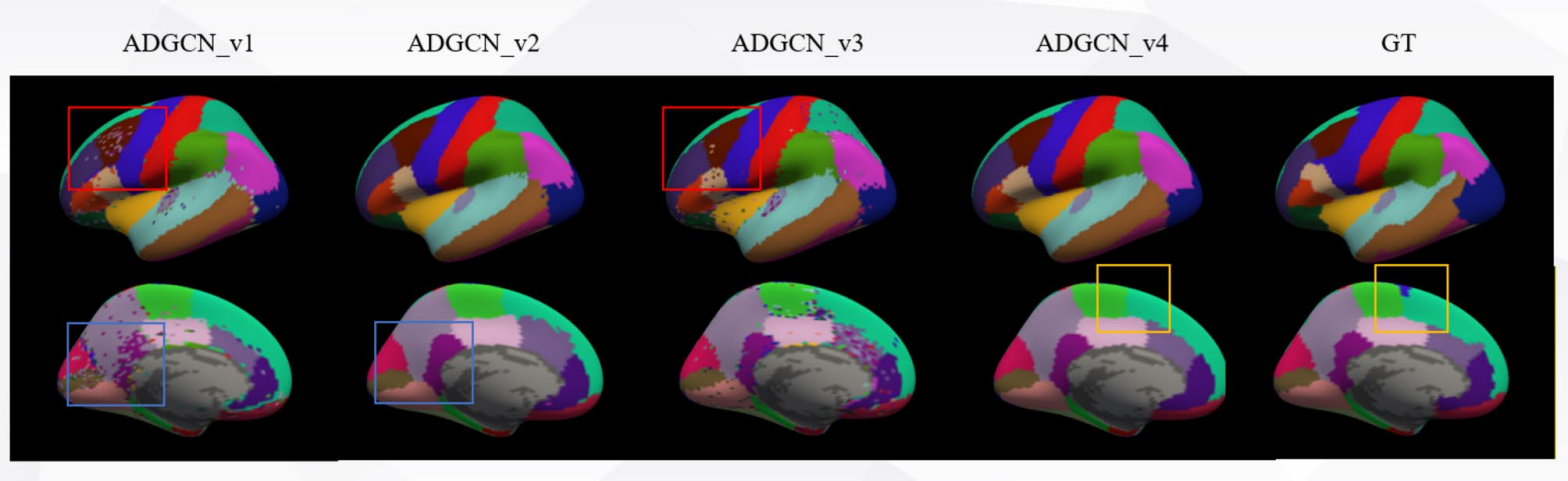
Effect of the number of hidden layers and SE module on parcellation performance. Note: ADGCN_v1: GCN network (1 hidden layer); ADGCN_v2: GCN network (7 hidden layer: 16, 32, …, 16); ADGCN_v3: GCN network (SE ration = 4, 1 hidden layer); ADGCN$\:\_$v4: GCN network (SE ration = 4, 7 hidden layers: 16, 32, …, 16).

The qualitative result of ADGCN is shown in Fig. 7, which shows the parcellation performance of randomly selected subjects. The inflated surface of the brain is covered with predicted labels. In the first layer of GCN, each node mainly aggregates the information of its direct neighbors and updates its representation based on this information. As the number of layers increases, GCN is able to indirectly aggregate the information of more distant neighbors. For example, in the second layer of GCN, the node contains not only the information of its direct neighbors, but also indirectly contains the information of the two-hop neighbors. This process can be continued, and the scope of information aggregation is expanded by one hop with each additional layer. Comparing ADGCN$\:\_$v1 and ADGCN_v2 in Fig. 7, it can be found that the increase in the number of hidden layers does bring about an improvement in segmentation performance, greatly alleviating the confusion of labels in brain regions and reducing segmentation anomalies (the second row of blue boxes). The SE module actually plays a role of feature screening by assigning weights to different channels. In GCN, this helps to reduce the impact of noise information and improve the robustness of the model. Comparing ADGCN$\:\_$v1 and ADGCN_V3, it can be found that the segmentation performance in some brain regions is indeed improved to a certain extent (the first row of red boxes). The combined use of the SE module and the U-shaped deep network, namely the ADGCN network, will greatly improve the segmentation performance, but the unclear boundaries of brain regions still exist (the yellow box in the second row).

**Fig. 8:**
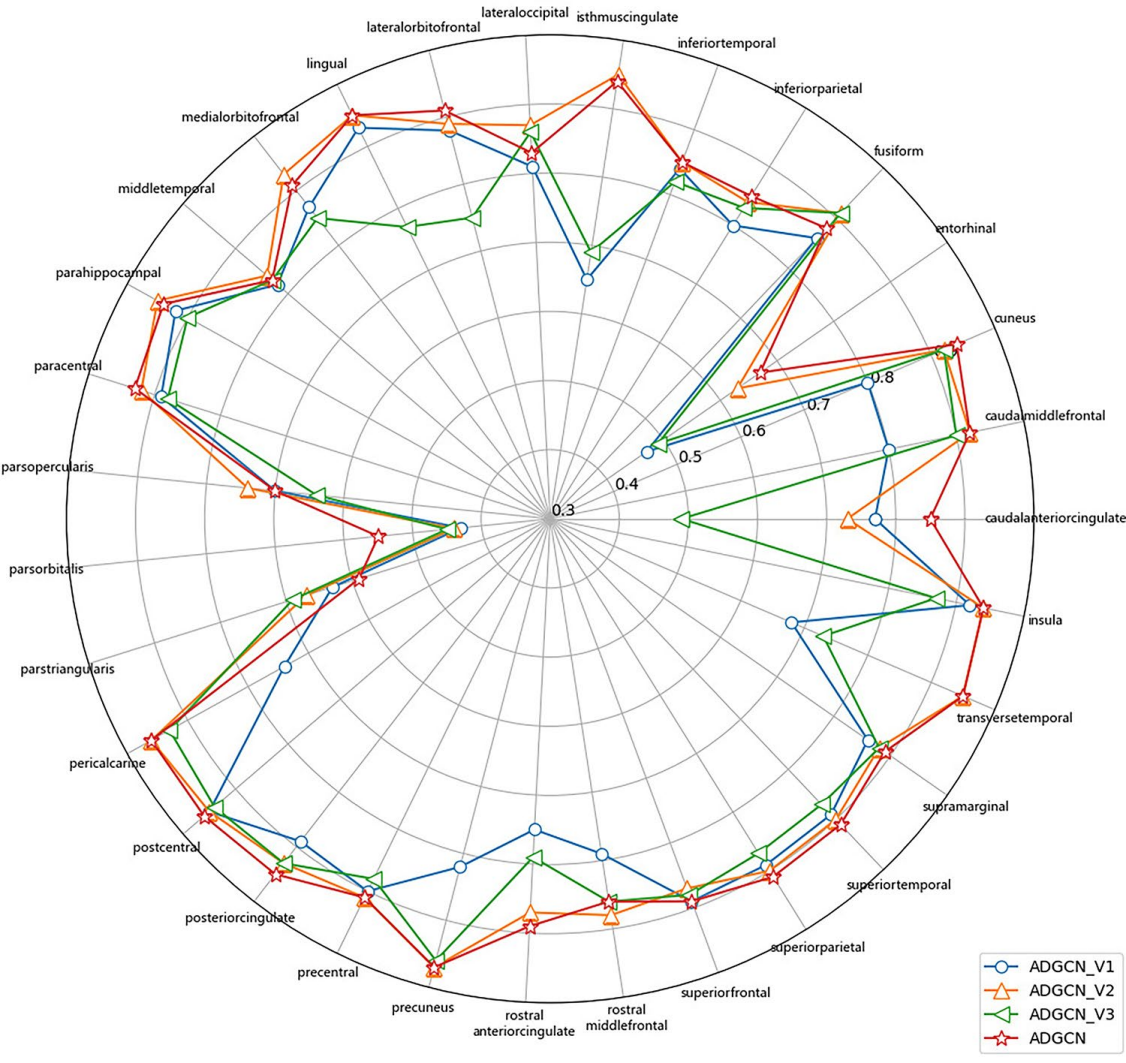
Dice coefficients of each brain region across different ADGCN model variants (ADGCN_V1, ADGCN_V2, ADGCN_V3, and ADGCN).

Figure 8 displays the Dice coefficients of each brain region across various ADGCN model variants (ADGCN_V1, ADGCN_V2, ADGCN_V3, and ADGCN). As shown in the figure, the parcellation performance of certain brain regions has indeed improved to some extent. Compared to ADGCN_V1, ADGCN_V2, and ADGCN_V3, the overall performance of ADGCN is more superior, with significant improvements in parcellation accuracy particularly in regions such as the Posteriorcingulate, Lingual, and Lateralorbitofrontal. Notably, the performance gains in the Parstriangularis and Caudalanteriorcingulate regions are especially pronounced, which indirectly reflects the positive impact of the introduction of the SE module and the increase in the number of hidden layers on model performance. However, in some brain regions, the performance of ADGCN is not as strong as that of other variants. For instance, in the Parstriangularis region, the parcellation performance of ADGCN is relatively poor. This may be attributed to the complex structure of the Parstriangularis region, which is characterized by ambiguous boundaries and high morphological variability. The SE module in ADGCN, while effective in capturing global contextual information, may overly rely on such global features, potentially neglecting local details and leading to reduced segmentation accuracy in this region. Additionally, although the increased number of hidden layers in ADGCN enhances the model’s expressive capacity, it may also introduce the risk of overfitting, particularly when the training data distribution is imbalanced or the sample size is insufficient. This could limit the model’s generalization ability for certain specific brain regions. Therefore, future work could focus on optimizing the weight allocation mechanism of the SE module or incorporating more refined feature extraction strategies to further enhance the model’s parcellation performance in complex brain regions.

The performance of ADGCN compared with other methods is shown in Table 3. Spherical domain method: FreeSurfer software^22^was used to segment the cerebral cortex surface, achieving Dice score of 83.69 and accuracy rate of 77.74. As a classic cortical surface segmentation tool, FreeSurfer can automatically extract cortical surfaces and segment them, but rely on traditional geometric and statistical methods, it is difficult to deal with complex nonlinear features. SPHARM-Net^36^ proposed a new spherical harmonic CNN based on the convolution theorem, which is used to simulate full-bandwidth convolution and maintain rotational equivariances while avoiding the specific neighborhood definition of spherical convolution. dice coefficients of 88.64% and 85.45% were obtained in Mindboggle and NAMIC, respectively. However, the dependence of this method on spherical parameterization may limit its applicability and have high computational complexity.

Deep Convolutional neural network (DCNN)^19^establishes a highly nonlinear mapping from the shape features of the cortex to the segmentation labels without the need for cortical surface registration, obtained 86.18% Dice coefficient. This method does not require registration and is suitable for processing infant brain data (with large individual differences), but CNNs are limited in their performance when processing non-regular mesh data and may not adequately capture the complex topology of the cortical surface. The Spherical U-Net (SU-Net)^18^optimized with the new features on the cortical envelope boundary obtained 86.59% Dice coefficient. This method uses the spherical registration method to calculate the deformation field to generate the deformation geometric features that are most consistent with the real ground block boundary and to overcome the dipole moment change of rotational invariance. However, the calculation complexity of spherical convolution is high and requires high data preprocessing (such as spherical parameterization). SegRecon^35^combined reconstruction and segmentation of cortical surfaces directly from MRI volume, trained a volume-based neural network to predict the signed distance of each voxel to multiple nested surfaces and its corresponding spherical representation in the atlas space, the dice coefficient of 88.69% is obtained. This method jointly learns surface reconstruction and segmentation tasks, which can optimize two goals at the same time, but has high requirements for computing resources. Attention-gated spherical U-net^34^applied the attention mechanism to the segmented spherical U-net of fetal cortex and obtained 89.90% dice coefficient. The Dice coefficient of 90.46% was obtained by Spherical-deformable U-Net (SDU-Net)^40^, which can be adapted to locate cortical structures of different sizes and shapes. This method combines the multi-scale feature extraction capability of U-Net and the geometric perception capability of spherical convolution, and the dependence on spherical mapping may introduce distortion. Zhao et al.^39^made use of the spherical topological properties of the cortical surface and use the spherical network as an encoder to extract higher cortical features. Then, two special decoders are built specifically for the registration and segmentation tasks. In order to extract more meaningful spatial features, a novel segmentation graph similarity loss is also designed to exploit the relationship between registration and segmentation tasks, and the dice coefficient of 92.70% is obtained by this method. Original domain: GCN^51^directly segments the cerebral cortex onto the primitive cortical surface manifold without needing spherical mapping, learns the mapping relationship between the cortical attribute pattern on the primitive surface manifold and the segmentation label, and obtains 86.48% Dice coefficient. However, this method relies on the processing of local blocks and may ignore global context information. End-to-end deep cortical surface parcellation network — Deep Brain Cortical Parcellation Network (DBPN)^52^, using a U-shaped rough segmentation network and a fine network for fine-tuning rough segmentation, the Dice coefficient of 84.60% is obtained. The network guarantees the smoothness of the parcellation scheme on the surface, but only uses the information based on the Euclidean coordinate system. ASEGAT^44^consists of two graph attention modules and a SE module, and introduces anatomical constraint loss. This network improves the segmentation performance of the network and the consistency of regional adjacency relations, and obtains 89.00% dice coefficient and 90.65% accuracy. This method relies on anatomical constraints, which may limit its ability to generalize on other tasks or datasets. The Spectral GCN (SGCN)^53^ convolutional network for cortical segmentation overcomes the difficulty of directly learning surface data across different surface bases, achieving an Dice coefficient of 85.37% and an accuracy rate of 86.97%. The spectral embedding method has high computational complexity and may not be suitable for large-scale data processing. Current spectral convolution methods deal with this variance by aligning feature vectors with reference brains separately in slow iterative steps, however as a global representation, the spectral features used in this approach can lose subtle local information.

By observing Table 3, it is found that compared with the cortical surface parcellation directly on the original domain, the methods based on spherical domain have achieved better performance, which may be due to the complex structure of the cerebral cortex and the large differences between individuals. The corresponding relationship between the map and individuals can be better established with the help of the topological isomorphic mapping sphere. Therefore, this kind of parcellation methods in the sphere domain have obtained better performance. For example, the method proposed by Zhao et al.^39^is the best in parcellation performance. It mainly makes use of the time consistency of the longitudinal cortical surface, the inherent constraints of shared space and time feature representation, and performs cortical feature extraction, registration and segmentation on the sphere domain. This results in significant improvements in both registration/segmentation accuracy and longitudinal consistency, especially in small and challenging cortical areas. However, the performance of spherical-domain based methods depends heavily on accurate cortical surface registration. This method of segmentation in the spherical domain has the following two disadvantages: First, the operation of mapping the surface of the cerebral cortex to the spherical surface is complex and time-consuming. Second, the spherical mapping of the surface of the primitive cortex is inherently sensitive to topological noise and cannot handle damaged brains that violate the spherical topology^54^. Therefore, the segmentation method based on spherical domain is not suitable for patients with neurological diseases.

**Table 3:** Performance comparison of ADGCN with other methods.

Methods	Operation Space	Datasets	Dice (%)	Accuracy (%)
SPHARM-Net ^[36]^	Spherical domain	Mindboggle/NAMIC	88.64/85.45	-
FreeSurfer ^[22]^		Mindboggle	83.69	77.74
DCNN ^[19]^		Private Datsets	86.18	-
SU-Net ^[18]^		Private Datsets	86.59	-
SegRecon ^[35]^		Mindboggle/ABIDE/OASIS	88.69	-
You et al. ^[34]^		Private Datsets	89.90	
SDU-Net ^[40]^		NAMIC	90.46	-
Zhao et al. ^[39]^		BCP/OASIS	**92.70**	-
GCN ^[51]^		Private Datsets	86.48	-
DBPN ^[52]^	Original domain	Mindboggle	84.60	-
SGCN ^[53]^		Mindboggle	85.37	86.97
ASEGAT ^[44]^		Mindboggle	89.00	**90.65**
ADGCN (Proposed)		Mindboggle	88.53	90.27

In the original domain, ASEGAT is the model with the best parcellation performance at present, which assigns different attention coefficients to each neighbor node, but may make the model pay more attention to the relationship between local nodes, which increases the complexity and computation of the model. Although ADGCN is not as good as ASEGAT in parcellation performance, it makes direct use of graph structure information through convolution operation, which makes the use of graph structure information relatively direct and efficient, making the training process relatively stable and easy to converge to a better solution. Therefore, both GCN and GAT have advantages and disadvantages, and we can choose different models according to the complexity of graph structure and computing resources. In some cases, GCN and GAT can be combined to take advantage of the best of both worlds. For example, GCN can be used for initial feature extraction and aggregation, followed by GAT for further feature fusion and classification. The proposed model also has insufficient, firstly, the intrinsic structural information of the cortical surface and the global information of the brain region are not fully utilized. For example, the sMRI image was resampled to the standard space to achieve the consistency of node number and structure in this study, but the structural information may be lost and inaccurate, and it is also not conducive to the introduction of graph features and the construction of heterogeneous graph, which may affect the overall performance of parcellation. Therefore, under the premise of low memory consumption, we recommend calculating the initial sampling rate based on local structural complexity or aligning the sMRI images to the standard space using non-rigid registration methods (such as deep learning-based registration algorithms) to reduce the loss of structural information. Second, our proposed method is highly sensitive to the quality of the surface reconstruction step, for example, small errors or holes in the reconstructed cortical grid may make the graph convolution method unable to complete the segmentation task. Therefore, we propose to develop a topology-preserving iterative optimization algorithm in subsequent research. This algorithm will progressively correct mesh errors while strictly maintaining the integrity of cortical topological structures, enabling automatic repair of cortical meshes with minor errors or holes, thereby effectively mitigating the impact of surface reconstruction quality on parcellation accuracy. Finally, we only utilized surface-based morphological features of surface (e.g., volume, thickness, area, and gyrification), lacking the capacity to reflect microstructural organization and functional characteristics, which may limit segmentation accuracy and anatomical detail recognition. To address these limitations, we propose to implement a Riemannian geometry-based cortical folding quantification method in future work, enabling the extraction of dynamic cortico-subcortical structural connectivity features and construction of multi-timepoint developmental trajectory models. In addition, the combination of voxel-based morphological and Surface-based morphological has the potential to improve the accuracy of detecting morphological changes in the cortex, helping researchers and clinicians better understand normal neurobiological processes in the brain. Therefore, we plan to use DARTEL for high-precision registration, calculate gray matter density (GMD) and gray matter volume (GMV), and then extract local volume change features. Finally, we use graph neural network to integrate voxel-based and surface-based morphological features. While the current study primarily focuses on healthy subjects, we acknowledge that different datasets may present distinct characteristics and challenges, and cortical features may vary across populations. To enhance the generalizability of our method and explore its clinical diagnostic potential, we plan to validate our approach on the Autism Brain Imaging Data Exchange (ABIDE) datasets, which are larger, more diverse, span a wider age range, and include both individuals with autism spectrum disorder (ASD) and healthy controls.

## Conclusion

This study proposes an attention-guided deep graph convolutional brain surface data learning framework, ADGCN, for end-to-end parcellation of cortical surfaces. The model incorporates a deep graph convolution layer with a symmetrical U-shape architecture, which facilitates the preservation and propagation of features from the original brain graph while effectively capturing its patterns. complex structure. Furthermore, the integration of squeeze and excitation modules enable the network to dynamically adjust the feature response across channels, enhancing sensitivity to important features and suppressing less relevant ones. Experimental evaluations on the Mindboggle datasets demonstrate the validity and interpretability of the proposed method in segmenting primitive cortical surface manifolds, offering potential improvements in the diagnostic accuracy of neurological disorders and the objectivity of therapeutic outcome assessments.

## Data Availability

The data supporting the findings of this study are openly available in Mindboggle at https://mindboggle.info (DOI: 10.3389/fnins.2012.00171).
